# Religious Conspiracy Theories About the COVID-19 Pandemic Are Associated With Negative Mental Health

**DOI:** 10.3389/ijph.2022.1604324

**Published:** 2022-08-26

**Authors:** Alice Kosarkova, Klara Malinakova, Lukas Novak, Jitse P. Van Dijk, Peter Tavel

**Affiliations:** ^1^ Olomouc University Social Health Institute (OUSHI), Olomouc, Czechia; ^2^ Department of Community and Occupational Medicine, Faculty of Medical Sciences, University of Groningen, Groningen, Netherlands; ^3^ School Kosice Institute for Society and Health, P.J. Safarik University in Kosice, Kosice, Slovakia

**Keywords:** mental health, COVID-19 pandemic, religious conspiracy beliefs, spirituality, religiosity, religious coping

## Abstract

**Objectives:** Together with the COVID-19 pandemic, conspiracy theories have begun to spread. Evidence is lacking for religious conspiracy theories (RCT) related to COVID-19 in a non-religious environment. This study aimed to assess links between religiosity and spirituality (R/S) and RCT about COVID-19, and to examine their associations with mental health.

**Methods:** A sample of Czech adults (*n* = 1,273, mean age = 47.5, SD = 16.4; 51.5% male) participated in the survey. We measured R/S, RCT, negative religious coping (NRC), feelings impairment and mental health symptoms.

**Results:** We found R/S were significantly associated with RCT with β 0.71 (95% confidence interval [CI] 0.59–0.82) for the strongest association. Moreover, RCT and NRC were strongly associated with paranoia, anxiety and depression. The most frequent association was found for NRC and paranoid ideation, with β of 0.35 (95% CI 0.26–0.44).

**Conclusion:** Our findings showed associations between religiosity/spirituality and beliefs in religious conspiracy theories about COVID-19. Moreover, these RCT and negative religious coping were linked to higher possibility of mental health problems. Understanding these associations may help prevent this negative impact and contribute to the effectiveness of psychotherapeutic help.

## Introduction

When the World Health Organization (WHO) declared the coronavirus disease COVID-19 as a pandemic in March 2020 [1], the whole world began to face a real challenge. Hand in hand with the number of infections and enhanced by social media, the number of false reports and misinformation increased and spread as fast as the virus itself, so that in addition to the pandemic, the world also began to fight an “infodemic” [2]. Subsequently, the pandemic has been accompanied with anxiety, fear or stress that can have a detrimental impact on mental health [3, 4]. These negative psychological outcomes can consequently have an adverse effect on the immune functioning and can increase susceptibility to disease [5, 6]. Such outcomes may become a barrier to effective physical and mental health interventions [7].

In a period of uncertainty, misinformation and feelings of powerlessness, people are likely to be attracted to conspiracy theories [8, 9], which can be seen as attempts to explain inexplicable events as a secret plot of multiple powerful actors working together [10]. According to Douglas [11], conspiracy theories appear to provide broad explanations and to satisfy important motives that can be characterized as epistemic (e.g., a need to understand and to gain a subjective certainty), existential (e.g., an urge for security and control) and social (e.g., a desire to maintain a positive image of the self or a group). Thus, it is obvious that as COVID-19 turned into a worldwide issue with misinformation drowning out credible sources of information, various theories have also rapidly spread [12, 13]. The studies of [14] showed that among the strongest predictor of beliefs in COVID-19 conspiracy theories are predispositions to view events as products of conspiracy thinking, denialism and to reject expert information. Such beliefs were also found to be associated with political motivation and ideological factors [14, 15]. It was also found that beliefs in conspiracy theories may be linked to negative health and adverse social effects [10, 12]. People who believe in conspiracy theories are more likely to develop negative attitudes toward health recommendations, are not willing to stick to them and do not cooperate in reducing infection rates [15–17].

Religiosity and spirituality (R/S) represent important dimensions of the lives of many people and an important resource for health and well-being [18]. A large number of studies have shown positive associations between R/S and human health [18, 19], among them also connections with a lower occurrence of depressive symptoms and anxiety [20]. Similarly, both R/S are often studied as a source of resilience and coping. Coping can be seen as a process of trying to manage the balance between the demands of the threatening situation and the accessible sources. One such a source may be religion [21], which could offer different ways of coping with different situations [22]. Pargament [23] defined religious coping as efforts to understand and deal with life stressors in ways related to the sacred. The term sacred includes traditional ideas about God, divinity or higher powers, as well as other aspects of life associated with divinity or divine qualities [24]. A positive effect of religious coping, i.e., protecting individuals from mental health problems when dealing with difficulties, has been shown in many studies (e.g., [25, 26]). However, this protective effect was associated with coping which reflects a secure relationship with God, a spiritual connectedness with others and a benevolent worldview [27]. Negative religious coping (NRC) reflects a spiritual tension and struggles within oneself, others and the divine [27]. It is connected to a punishing God’s reappraisal when dealing with negative and stressful life situations [28]. When NRC strategies were used, the outcomes were opposite and associated with worse psychological adjustment [27, 28], higher levels of depression and isolation [21], worse quality of life and life satisfaction [29], higher risk of suicide [29, 30] and a higher risk of mortality [31].

Furthermore, some studies have shown associations of R/S with conspiracy beliefs [32, 33]. Conspiracy beliefs are strongly related to partisan and ideological motivations [14, 34] and for some people their individual’s R/S network plays a role in the endorsement of conspiracies and may facilitate one’s attraction to a conspiracy [33]. Therefore, when difficult-to-understand phenomena are explained in the context of religion or incorporated into a broader belief system [33], religious conspiracy theories (RCT) can emerge. In addition, people within a similar R/S mindset have a social network in which such theories can be mutually supported by like-minded people and thus can spread very effectively [35]. Consequently, it is possible that people with beliefs in RCT theories may tend to have negative views of their difficulties, impaired feelings and worse mental health [36, 37].

The Czech Republic is characterized by a high degree of secularization, as most people do not report any religion affiliation or regular church attendance [38]. Recent research in the field of conspiracy theories [14, 15, 34] suggest that psychological issues such mental health and R/S may predispose to conspiracies. However, we may also assume that R/S and NRC may be linked to beliefs in RCT abour COVID-19 and that negatively perceived R/S and religious conspiracy beliefs may be further associated with poorer mental health outcomes during pandemic [10, 29]. Thus, our study would like to explore this area in a secular setting and add a different perspective to existing knowledge. Assessing the links between R/S and religious conspiracy beliefs and their associations with negative emotions, paranoid ideation, anxiety and depression in a secular country can bring interesting findings and may help us to understand how these variables affect mental health.

Therefore, the aim of this study is to assess the associations of religiosity/spirituality and negative religious coping with beliefs in religious conspiracy theories about COVID-19 and to examine the associations of such beliefs and coping on mental health by assessing their relationship with emotional impairment, paranoia, depression and anxiety during the first wave of the COVID-19 pandemic.

## Methods

### Participants and Procedure

For this study we used data from the Czech population aged 18 to 97. The data was collected during the first lockdown in April 2020 through an anonymous online survey aimed at depicting the actual situation through the most critical time of the first wave of the COVID-19 pandemic. The online survey was prepared at the researchers’ institution and a professional agency ensured its distribution in order to achieve a balanced sample regarding age and sex. Details on age and gender regarding the composition our sample vs. a Czech national representative sample can be found in the Appendix (see Supplementary Materials).

Consequently, in order to ensure the high quality of the data, the following data were excluded: 1) an extremely short time filling in the survey (i.e., less than 10 min for a survey that typically lasted around 45 min); 2) a unified pattern of responses, i.e., responding to most of the items in the survey in the same way. After exclusion of the problematic subjects (*n* = 13) the final sample consisted of 1,273 respondents (mean age = 47.5, SD = 16.4; 51.5% male).

At the beginning of the survey, participants received written information on the aim of the study and the anonymized handling of data and were made familiar with the system. Participation in the survey was fully voluntary, with the possibility of leaving the study at any time before or during the survey without giving reasons. Respondents had to explicitly express their informed consent with participation prior to the study. The study design was approved by the Ethics Committee of the Palacký University, Olomouc (No. 2020/06).

### Measures

Religious coping was assessed using the negative religious coping subscale (NRC) of the Brief RCOPE [27]. The instrument was validated for the Czech condition [39]. It is composed of seven items that reflect a less secure religious relationship growing out of a tenuous and ominous view of God or the world. Items are rated on a seven-point scale, with possible answers ranging from “not at all” [1] to “a great deal” [4], and the total score ranges from 7 to 28. For further categorizations of responses, each of the item scores was dichotomized according to the approach of Fitchett [40]. Scores of 1 or 2 were recoded to “0” (did not use NRC) and scores of 3 or 4 recoded to “1” (used NRC). Consequently, when any of the seven NRC items was “1,” a respondent was classified as showing NRC [41]. Cronbach’s alpha was 0.84 in our sample.

Religious conspiracy theories were assessed using four statements capturing common religious opinions on the COVID-19 pandemic. The statements were generated from searching the Internet and social media during the first weeks of the pandemic. Although the approach may not be completely exhaustive, we tried to capture the most common theories involving religious themes. The assessed statements were: “The current coronavirus pandemic is God’s punishment”; “The current pandemic is a punishment for the moral decline of the Church and for the liberal attitudes of Pope Francis”; “The current pandemic has been foretold by some religious visionaries”; and “The current pandemic is only the beginning of the events described in the book of the Apocalypse.” Participants were asked to mark to what degree the information corresponds to the truth. Possible options ranged from “does not correspond at all” (0) to “definitely corresponds” [3]. When any of the statements was marked as “corresponding” [2] or “definitely corresponding” [3], the respondent was classified as believing in RCT.

Negative feelings impairment was assessed by the question: “In connection with the pandemic, has anything changed in your life in the following areas?” These areas were: the feeling of loneliness, threat, fear and anxiety, helplessness, loss of hope. The possible answers were [1]: “worsened” [2]; “unchanged” [3]; “improved” and [4] “the question does not concern me.” For the purpose of further analysis, the answers for each item were dichotomised. Respondents who answered 1 (worsened) were classified as experiencing negative feelings impairment.

Paranoia was assessed using the Paranoid Ideation subscale of the Brief Symptom Inventory (BSI-53) [42, 43]. The introductory instruction was: “How much has the following symptoms problem distressed or bothered you during the past month?” It was followed by items rated on a five-point Likert scale, with possible answers ranging from [1] “not at all” to [5] “extremely.” For the purpose of further analysis, the subscale was dichotomized following the approach of Stewart [44], i.e., the summary score of the answers was computed and participants with a score of 66 or higher were considered as paranoid, and the rest as non-paranoid. Cronbach’s alpha for the subscale in our sample was 0.83.

Anxiety was measured using the Overall Anxiety Severity and Impairment Scale (OASIS) [45]. It is a 5-item self-reported scale that assesses the severity of anxiety as well as behavioural and social avoidance caused by anxiety symptoms. Respondents were instructed to endorse the response that best describes their experiences over the past week. In our study, we used a shortened version with abbreviated responses [46] that ranged from [1] “never” to [5] “all the time.” In the main analyses, participants’ responses were dichotomised in the following way: items 4 (often) and 5 (all the time) were recoded as “1” (anxious), and items from 1 (never) to 3 (sometimes) “0” (non-anxious). Cronbach’s alpha in our sample was 0.89.

To assess depression, we used an abbreviated version the Overall Depression Severity and Impairment Scale (ODSIS) [47]. This self-reported scale assesses the severity and functional impairment associated with depressive symptoms as well as its impact on work and social life. The respondents choose from responses on a 5-point Likert-type scale ranging from [1] “never” to [5] “all the time.”. For further analyses, the answers were dichotomized thus: items 4 (often) and 5 (all the time) were recoded as “1” (depressed), and items from 1 (never) to 3 (sometimes) were recoded as “0” (not depressed). In the present study, Cronbach’s alpha in our sample showed a high consistency with α = 0.92.

Spirituality was measured using the Daily Spiritual Experience Scale (DSES). The scale measures the frequency of ordinary experiences of connection with transcendence in everyday life [48]. The present study used an adapted 15-item version of the scale validated for the Czech environment [49]. Each item was evaluated on a six-degree Likert scale graded according to the intensity of experiencing the observed phenomena, ranging from “never” [1] to “many times a day” [6]. For the analysis, we treated the total score as a continuous variable. Cronbach’s alpha for the whole scale has an excellent internal consistency, with α = 0.96 in our sample.

Religiosity was measured using the following question: “At present, would you call yourself a believer?” with possible answers: “yes, I am a member of a church or religious society”; “yes, but I am not a member of a church or religious society”; “no”; “no, I am a convinced atheist”. For the purpose of further analysis, participants who reported “yes” were dichotomized as religious.

Sociodemographic characteristics, such as sex, age, education level, marital status and economic activity, were obtained by means of the questionnaire.

### Statistical Analyses

In the first step, the descriptive statistics of the key study variables were calculated. The differences in basic characteristics and in the observed categorical variables were assessed using the Chi-Squared test. In the further step, we conducted Principal Component Analyses (PCA) on items for each scale. In the next step, component scores were calculated from the first extracted component. These scores were used in the further analyses. Prior to further analysis, all continuous dependent and independent variables were standardized to Z-scores. We assessed the associations of spirituality, religiosity and NRC with the whole RCT using linear mixed model, i.e. multiple linear regression. Logistic regression was used only to asses changes in feelings associated with COVID-19 pandemic. In all regression models, we set a time needed for finishing the questionnaire as a random effect. In each regression analysis, we used a model with random intercept. If chi-square difference test suggested that a model with random slope has a better fit, we included a random slope into a model. Subsequently, the procedure was repeated for the associations of RCT and NRC with changes in life during the pandemic as well as with paranoia, anxiety and depression. The *p*-values of these univariate analyses were corrected for the family-wise error rate (FWER) using a Bonferroni approach. All multiple regression models were first assessed as crude and consequently adjusted for sex, age, and the highest level of education. The data have been adjusted to partial out variance of variables which might confound our results. This approach helped us to interpret the data for the given variable without the constraints of removed variables. Each of the independent variables was assessed in a separate model. All analyses were performed using the statistical software IBM SPSS version 25 (IBM Corp., Armonk, NY, United States) and R (Version 4.0.3; R Core Team, 2020).

## Results

### Description of the Population

The sociodemographic characteristics of the sample are presented in Table 1. The sample represents the Czech population aged 18 years and older (mean age = 47.5; SD = 16.4; 51.5% male). Of the whole sample, 342 respondents reported some kind of RCT belief and 131 religious participants reported NRC. Regarding sex, the comparison of groups revealed significant difference (*p* < 0.05) only for the respondents believing in NRC. Moreover, the respondents differed significantly in levels of education (*p* < 0.001 for RCT, *p* < 0.05 for NRC), and a comparison showed significant difference among religious respondents within the group believing in RCT(*p* < 0.001) and within the group using NRC (*p* < 0.01).

**TABLE 1:** Description of the study population, total and by RCT and NRC (The COVID-19 online survey, the Czech Republic, 2020).

	Total		Religious conspiracy theory belief^a^		p-Value	Negative religious coping^b^		p-Value
	N	%	N	%		N	%	
Sex								
Male	655	51.5	158	46.2	*p* < 0.05	63	48.1	n.s.
Female	618	48.5	184	53.8		68	51.9	
Age								
18–29 years	125	16.9	54	15.8	n.s.	18	13.7	n.s.
30–44 years	371	29.1	102	29.8		35	26.7	
45–59 years	312	24.5	80	23.4		30	22.9	
60–99 years	375	29.5	106	31.0		48	36.6	
Marital status								
Single/Divorced/Widow(er)	427	33.5	181	52.9	n.s.	66	50.4	n.s.
Married/Partner relationship	846	66.5	161	47.1		95	49.6	
Education								
Elementary	109	8.6	37	10.8	*p* < 0.01	9	6.9	*p* < 0.05
Secondary vocational	559	43.9	169	49.4		71	54.2	
Secondary with graduation	408	32.1	94	27.5		30	22.9	
College	197	15.5	42	12.3		21	16.0	
Economic activity								
Employee	637	50.0	165	48.2	n.s.	53	40.5	n.s
Self-employed	64	5.0	21	6.1		7	5.3	
Household^c^/unemployed	118	9.3	37	10.8		11	8.4	
Student	77	6.0	16	4.7		8	6.1	
Disabled/old-age pensioner	377	29.6	103	30.1		52	39.7	
Religiosity^d^								
Believer, church member	109	8.6	45	13.2	*p* < 0.001	47	35.9	*p* < 0.01
Believer outside the church	313	24.6	140	40.9		84	64.1	
Non-believer	680	53.4	127	37.1				
Convinced atheist	171	13.4	30	26.9				
Total	1273	100	342	26.9		131	10.3	

a Believing in at least one religious conspiracy theory.

b NRC > “quite a bit” in any of the 7 items; these descriptive statistics were calculated only from a subset of participants: those who filled in that they were religious.

c Including maternity leave.

d independently of church attendance; n.s., non-significant.

### Religious Conspiracy Theory beliefs

**FIGURE 1:**
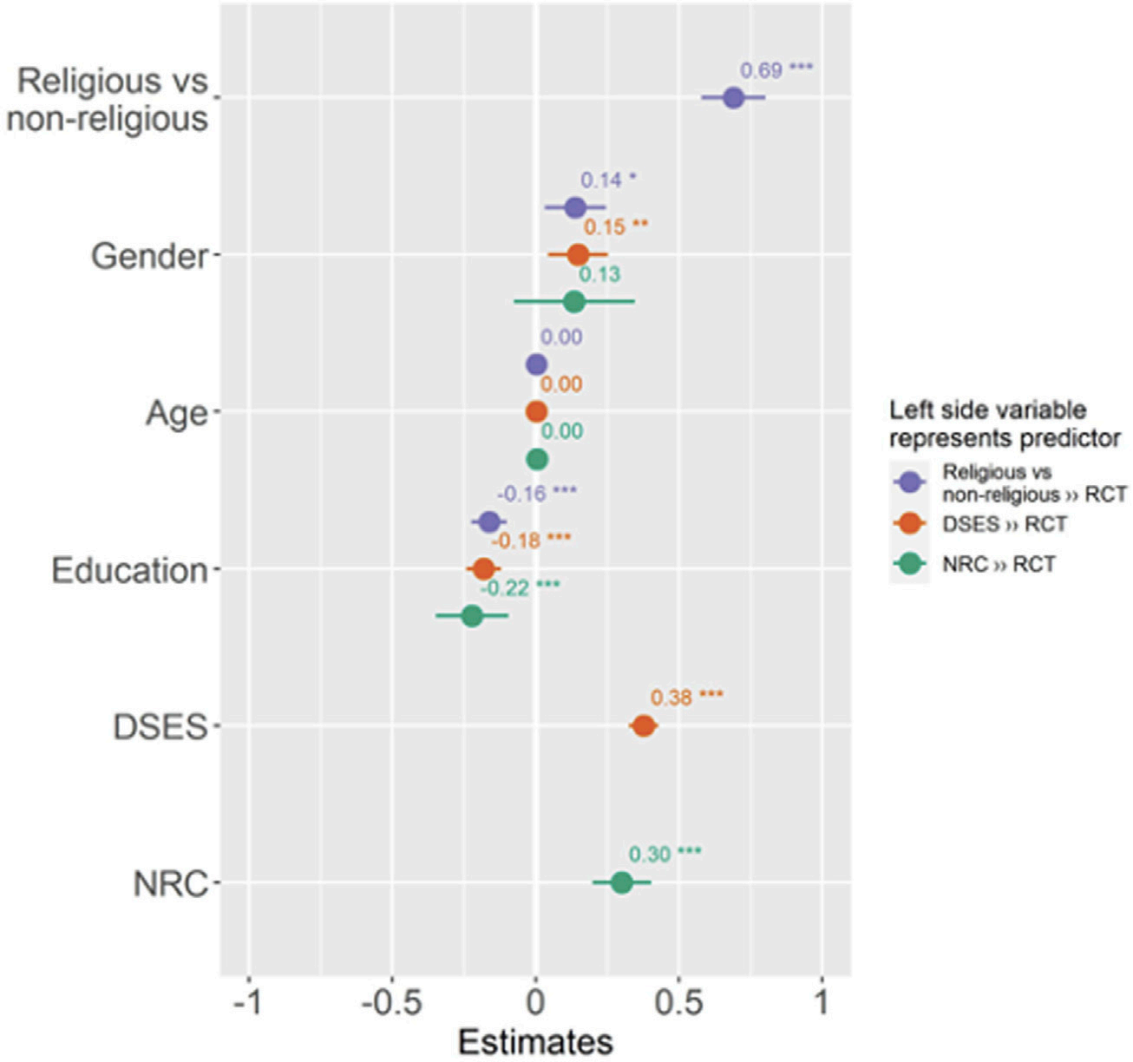
Forest plot depicting associations of religiosity, spirituality and negative religious coping with religious conspiracy theories. Figure also depicts an effect of variables which were statistically controlled (The COVID-19 online survey, the Czech Republic, 2020).

Table 2 shows the results of the binary logistic regression, crude and adjusted for sex, age, level of education and economic activity, aimed at the associations between spirituality, religiosity and NRC with RCT beliefs. Assessing religiosity, spirituality and NRC revealed that religiosity and spirituality are significantly (*p* < 0.001) associated with RCT beliefs around COVID-19. The results are also visually depicted in Figure 1.

**TABLE 2:** Associations of spirituality (standardized to Z-scores), religiosity, and negative religious coping with religious conspiracy beliefs core and adjusted for age, gender, and the highest level of education (beta coefficients and 95% confidence intervals) (The COVID-19 online survey, the Czech Republic, 2020).

		RCT sum
Religious	Crude	0.71 (0.59–0.82)***
vs non-religious	Adjusted	0.69 (0.58–0.80)***
Spirituality	Crude	0.38 (0.32–0.43)***
	Adjusted	0.38 (0.33–0.43)***
NRC	Crude	0.31 (0.20–0.41)***
	Adjusted	0.30 (0.20–0.40)***

Notes: RCT, religious conspiracy theory; NRC, negative religious coping.

*p < 0.05, **p < 0.01, ***p < 0.001; Bold text: significant after FWER correction.

### Feelings Impairment

Table 3 depicts the associations of RCT and NRC with the deterioration of feelings during the COVID-19 pandemic. After FWER correction, we found no significant associations for RCT and feelings impairment. However, associations were found between NRC and impaired feelings of threat and anxiety.

**TABLE 3:** Associations of religious conspiracy theories and negative religious coping with feelings impairment, both crude and adjusted for age, gender, and the highest level of education (odds ratios and 95% confidence intervals) ((The COVID-19 online survey, the Czech Republic, 2020).

		Loneliness	Threat	Fear and anxiety	Helplessness	Loss of hope
RCT	Crude	1.03 (0.89–1.19)	1.01 (0.90–1.14)	1.10 (0.98–1.25)	0.99 (0.87–1.13) 0.99 (0.86–1.14)	1.02 (0.85–1.23) 1.01 (0.84–1.22)
	Adjusted	1.03 (0.88–1.20)	1.02 (0.90–1.15)	1.09 (0.96–1.24)		
NRC	Crude	1.25 (1.00–1.56)	1.29 (1.06–1.58)* 1.33 (1.09–1.64)**	1.26 (1.03–1.54)* 1.31 (1.06–1.62)*	1.26 (1.03–1.55) 1.33 (1.07–1.66)	1.36 (1.05–1.78)
	adjusted	1.30 (1.03–1.64)				1.39 (1.06–1.83)

Notes: RCT, religious conspiracy theory; NRC, negative religious coping.

### Paranoia, Depression and Anxiety

**FIGURE 2:**
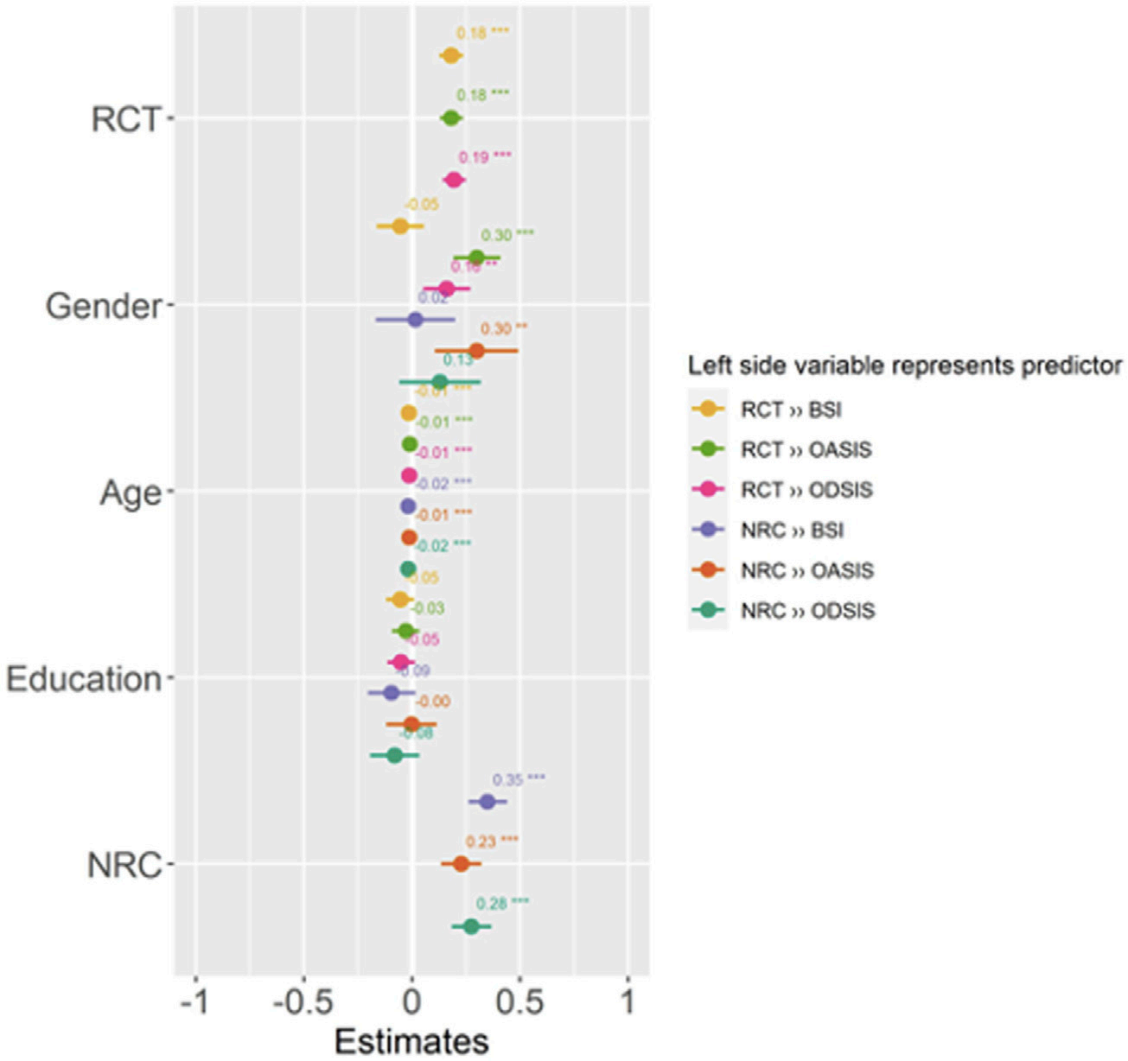
Forest plot depicting associations between religious conspiracy theories, negative religious coping and mental health linked to COVID-19 pandemic. Figure also depicts an effect of variables which were statistically controlled (The COVID-19 online survey, the Czech Republic, 2020).

The results of binary logistic regression assessing the RCT and NRC with paranoia and anxiety and depression frequency and intensity are presented in Table 4 and visually depicted in Figure 2. The results showed that both RCT and NRC were associated with higher paranoia, anxiety and depression. With every one unit increase in SD of the NRC, there was 0.35 increase in SD of the paranoia score in the adjusted model. Similarly, strong associations were found between RCT and paranoia, anxiety and depression. Specifically, one increase in SD of the RCT beliefs was associated with 0.28 increase of depression score in the adjusted model.

**TABLE 4:** Associations of religious conspiracy theories and negative religious coping with paranoia, depressions, and anxiety, both crude and adjusted for age, gender, and the highest level of education (beta coefficients and 95% confidence intervals) (The COVID-19 online survey, the Czech Republic, 2020).

		Paranoia	Anxiety	Depression
RCT	Crude	0.17 (0.12–0.23)***	0.19 (0.13–0.24) ***	0.20 (0.14–0.25) ***
	Adjusted	0.18 (0.13–0.24)***	0.18 (0.13–0.24) ***	0.19 (0.14–0.25) ***
NRC	Crude	0.35 (0.26–0.44) ***	0.22 (0.12–0.31)***	0.27 (0.18–0.37)***
	Adjusted	0.35 (0.26–0.44) ***	0.23 (0.13–0.32)***	0.28 (0.18–0.37)***

Notes: RCT, religious conspiracy theory; NRC, negative religious coping; *p < 0.05, ** *p* < 0.01 ***p < 0.001; Bold text: significant after FWER correction.

## Discussion

We assessed links between R/S and beliefs in RCT and examined the associations of such beliefs with mental health. RCT beliefs were found to be associated with both R/S and NRC. Although we found no associations of RCT and NRC with the impairment of negative feelings except associations of NRC with threat, fear and anxiety, both RCT and NRC were strongly associated with paranoia, anxiety and depression. The strongest association was observed between NRC and the beliefs in RCT about COVID-19.

The findings of higher RCT belief in the group with a secondary vocational education without graduation vary from the study of Klosfad at al. [50] which found a little effect of education on beliefs in conspiracy theories connected to Zika virus. We can assume that education can in some cases lead people to better argue against claims that seek to disprove their conspiratorial beliefs and to more effectively process information that supports their theories [50]. Nevertheless, our results are in line with results of studies showing the associations of education level and beliefs in conspiracy theories (e.g., [51, 52]). It is possible that more educated people are able to evaluate the credibility of information and their sources due to higher analytical thinking skills. In comparison, people without higher education may have a lower level of cognitive reasoning skills and awareness of counter-argumentation [52, 53].

Furthermore, we confirmed an expected association between both R/S with each of the assessed RCT beliefs. This is in line with a number of other studies showing the associations of R/S with conspiracy theories [32, 33], although these studies were not aimed directly at religious conspiracies. Moreover, the results revealed the association of RCT with religiosity was even stronger than with spirituality. In distinguishing religiosity and spirituality as two different concepts [54], it may be argued, that as religious people have some level of religious education and follow beliefs prescribed and taught by a particular institution [55], they may also be influenced by the way their specific religious group mobilises, debates and negotiates conspiracy theories [56]. From this point of view, such theories can be motivated by a desire to maintain a strong group identity [57]. Therefore, our results align with studies that showed that R/S identity [33] and ideological motivations [14, 34] may be predispositions of endorsement of conspiracy theories. Beliefs in RCT may also serve a religious group to defend their status quo or to define threats as demonic outsiders [56, 57]. Moreover, such beliefs can help with feelings of powerlessness and alienation and to explain incomprehensible things in the hostile world [58, 59].

In our study, we did not observe any significant associations of RCT beliefs with impaired feelings except feelings of threat, fear and anxiety. These findings are in contrast with the findings of other studies, which reported an increase of negative feelings during the pandemic in connection to conspiracy theories [61, 62] and to NRC [63]. It is possible that to understand and deal with COVID-19 pandemic situation, people use R/S as a source of relief from stress and mental suffering [60] and tend to use rather positive ways of coping that are usually used at the beginning of stressful events [64]. In addition, we can also assume the effect of a social desirability, which may reflect the effort to report religious coping strategies in accordance with social expectations, where reporting negative attitudes towards God could be considered morally unacceptable [65].

Our further results showed strong associations of NRC with paranoia. This finding is in line with the study of McConnell [66]. Paranoid individuals can be characterized by cognitive-perceptual biases of mistrust, preferentiality and intentionality and by a response to the perceived threat by guardedness, hostility or fear [67]. Therefore, paranoid ideation may also reflect the feeling of being abandoned and punished by the God or other people and reinforce the conflict with core beliefs and values [21, 27]. Such maladaptive NCR strategies may further influence one’s stability and underpin paranoid ideation.

Moreover, in our study, NRC was also associated with both anxiety and depression. These results correspond with the findings of studies that showed a relationship of NRC with depressive disorder [36, 37] and with anxiety [37, 41]. A possible interpretation could be that R/S discomfort and negative perception of God may lead to increased levels of anxiety and distress [68] and to a reduced experience of hope [37] and meaning of life [69]. Alternatively, anxious people may find the support from God or their church insufficient [70] and rather turn to negative ways of religious coping. Our results are in line with the findings of other studies that found connections between COVID-19 conspiracy beliefs and anxiety and depression [61, 71] and also extended the negative impact of conspiracies on mental health to the area of religious conspiracies. We suggest that in a desire to find explanations for unclear situations and to diffuse fear and uncertainty, people may believe in RCT which are linked to their R/S and the way they perceive the world around them. However, these beliefs further increase feelings of anxiety and powerlessness [62]. Consequently, people may also tend to believe in other conspiracies [57] and their anxiety or depression may be aggravated. Accordingly, beliefs in RCT can impair mental health and have an impact on further adjustment during the COVID-19 pandemic. As found that both NRC and beliefs in RCT are linked to negative mental health, our results support the findings of other studies [29, 62] suggesting that R/S and mental health may be prior to belief in specific conspiracy theories [14, 15, 34, 35]. Due to a cross sectional nature of our study, we were not able to conclude on causality, however, we treated negative religious coping as an independent variable, because we presumed that it can also reinforce mental health problems and we found associations with negative health outcomes, particularly with paranoia, depression and anxiety.

### Strengths and Limitations

This study has several important strengths. The first one is its large sample, which is balanced and close to having a national characteristic regarding age and sex. It is also one of the few studies exploring the relationship between RCT, R/S and mental health during the COVID-19 pandemic and describing significant associations in this area. Further, with its focus on specific area of religious conspiracies it contributes to other studies that found connections of COVID-19 conspiracy beliefs with anxiety and depression.

However, this study also has some limitations. The first is the cross-sectional design, which does not enable us to make decisive conclusions on the direction of causality. Thus, the present study should be confirmed by studies with a longitudinal design. Moreover, there could be a methodological limitation to our study as we assesses beliefs in RCT among all respondents, whereas NRC was assessed only among religious respondents. Thus, our design did not allow us to explore another possible model, i. e. to treat NRC as a dependent variable. Another limitation concerns the relatively modest number of religious respondents who reported NRC, which decreased the power of the study. However, this subsample still included 131 respondents. A further limitation can be the use of a self-report methodology, which can cause information bias and may be influenced by a social desirability. Nevertheless, in the area of assessing conspiracy theory beliefs, an online anonymous survey seems to be an applicable means of lowering the unwillingness of respondents to admit their true beliefs [72]. In addition, our measures may not have captured all relevant RCT known to the sample and the inferences about the associations might have been contingent on the specific conspiracy theories employed [34].

### Implications

Our results show that RCT beliefs concerning COVID-19 are related to an individual’s R/S and maladaptive NRC strategies. These findings may help to understand the factors influencing the dynamics of development of RCT and their associations with R/S areas of human lives. We also found that both RCT and NRC were negatively associated with mental health. This points out that some aspects of R/S may have a relevant impact on mental health and adjustment during the pandemic. This can be helpful for health care workers, as well as for workers in helping professions, such as psychotherapy, psychosomatic medicine, social work or pastoral care.

Further research should focus on the causal effects of the RCT beliefs dynamic and on the mutual interaction between R/S and conspiracies in general. It could also focus on a more specific categorization of the respondent groups according to their religiosity or spirituality and test for potential confounders between RCT, NRC and mental health.

### Conclusion

The impact of the COVID-19 pandemic is not only related to physical health but also involves psychological issues. Our findings emphasize the associations of religious conspiracy theories about the pandemic with R/S and NRC. The negative effect of RCT beliefs and of NRC was revealed by significantly higher levels of paranoia, depression and anxiety in those who reported such beliefs or/and a way of coping. Thus, this study offers a deeper understanding of the factors that might influence the development of religious conspiracy theories and contribute to studies on conspiracy theories and the extent to which these beliefs may affect mental health. Furthermore, it stresses the importance of addressing spiritual issues in order to minimize maladaptive coping strategies associated with RCT beliefs.
