# Corrigendum: Religious Conspiracy Theories About the COVID-19 Pandemic Are Associated With Negative Mental Health

**DOI:** 10.3389/ijph.2023.1605910

**Published:** 2023-04-12

**Authors:** Alice Kosarkova, Klara Malinakova, Lukas Novak, Jitse P. Van Dijk, Peter Tavel

**Affiliations:** ^1^ Olomouc University Social Health Institute (OUSHI), Olomouc, Czechia; ^2^ Department of Community and Occupational Medicine, Faculty of Medical Sciences, University of Groningen, Groningen, Netherlands; ^3^ School Kosice Institute for Society and Health, P.J. Safarik University in Kosice, Kosice, Slovakia

The authors neglected to include the funder “Sts Cyril and Methodius Faculty of Theology of Palacký University Olomouc, internal project Research Areas of Pedagogical Work with Children and Adults, IGA_CMTF_2022_004 to AK.”

The authors apologize for this error and state that this does not change the scientific conclusions of the article in any way. The first published version of the article has been updated.

