# Supplementary material for: Religious Conspiracy Theories About the COVID-19 Pandemic Are Associated With Negative Mental Health
Source: Int J Public Health. 2022 Aug 26;67:1604324. doi: 10.3389/ijph.2022.1604324 (PMC9458861; doi:10.3389/ijph.2022.1604324)
Supplement: Supplementary file 1 [file DataSheet1.PDF]

SUPPLEMENTAL APPENDIX

The COVID-19 online survey question wording
Czech Republic, 2020

(translated from the Czech language)

Negative Religious Coping

Please indicate to what do extent you experience the following states/thoughts?
(possible answers for each item are 1= not at all, 2 = a little bit, 3 = quite a lot, 4 = a great deal)

1. I wonder whether God had abandoned me.
2. I feel punished by God for my lack of devotion.
3. I wonder what I did for God to punish me.
4. Question God's love for me.
5. I wonder whether my church had abandoned me.
6. Decide the devil made this happen.
7. Question the power of God.

Religious conspiracy theories about COVID-19

In your opinion and according to your information, to what extent do the following statements correspond to the truth?
(possible answers for each item are 1= does not correspond at all, 2 = does not correspond, 3 = corresponds, 4 = definitely corresponds)

1. The current coronavirus pandemic is God's punishment.
2. The current pandemic is a punishment for the moral decline of the Church and for the liberal attitudes of Pope Francis.
3. The current pandemic has been foretold by some religious visionaries.
4. The current pandemic is only the beginning of the events described in the book of the Apocalypse.

Negative feelings impairment

In connection with the pandemic, has anything changed in your life in the following areas?
(possible answers for each item are 1= worsened, 2 = unchanged, 3 = improved, 4 = the question does not concern me)

1. Feeling of loneliness
2. Threat
3. Fear and anxiety
4. Helplessness
5. Loss of hope

Paranoia

How much has the following symptoms problem distressed or bothered you during the past month?

(possible answers for each item are 1= not at all, 2 = a little bit yes, 3 = moderately, 4 = quite strongly, 5 = extremely)

1. The feeling that others are responsible for most of your problems.
2. The feeling that most people can't be trusted.
3. The feeling that others are watching you or talking about you.
4. Feeling that others do not give you enough credit for your accomplishments.
5. The feeling that people will take advantage of you if you allow it.

Overall Anxiety Severity and Impairment Scale (OASIS)

The following items ask about anxiety and fear. For each item, select the answer that best describes your experience over the past week.

(possible answers for following items are 1 = never, 2 = infrequently, 3 = sometimes, 4 = often, 5 = all the time)

1. In the past week, how often have you felt anxious?
2. In the past week, how often did you avoid situations, places, objects, or activities because of anxiety or fear?

(possible answers for following items are 1 = none, 2 = mild, 3 = moderate, 4 = severe, 5 = extreme)

3. In the past week, when you have felt anxious, how intense or severe was your anxiety?
4. In the past week, how much did your anxiety interfere with your ability to do the things you needed to do at work, at school, or at home?
5. In the past week, how much has anxiety interfered with your social life and relationships?

Overall Depression Severity and Impairment Scale (ODSIS)

The following items ask about depression. For each item, select the number for the answer that best describes your experience over the past week.

(possible answers for following items are 1 = never, 2 = infrequently, 3 = sometimes, 4 = often, 5 = all the time)

1. In the past week, how often have you felt depressed?
2. In the past week, how often did you have difficulty engaging in or being interested in activities you normally enjoy because of depression?

(possible answers for following items are 1 = none, 2 = mild, 3 = moderate, 4 = severe, 5 = extreme)

3. In the past week, when you have felt depressed, how intense or severe was your depression?
4. In the past week, how much did your depression interfere with your ability to do the things you needed to do at work, at school, or at home?
5. In the past week, how much has depression interfered with your social life and relationships?

Daily Spirituality Experience Scale (DSES)

The list that follows includes items you may or may not experience. Please consider how often you directly have this experience, and try to disregard whether you feel you should or should not have these experiences. A number of items use the word ‘God.’ If this word is not a comfortable one for you, please substitute another word that calls to mind the divine or holy for you.

(possible answers for each item are 1= never, 2 = once in a while, 3 = some days, 4 = most days, 5 = every day, 6 = many times a day)

1. I feel God’s presence.
2. I experience a connection to all of life.
3. During worship, or at other times when connecting with God, I feel joy which lifts me out of my daily concerns.
4. I find strength in my religion or spirituality.
5. I feel deep inner peace or harmony.
6. I ask for God’s help in the midst of daily activities.
7. I feel guided by God in the midst of daily activities.
8. I feel God’s love for me, directly.
9. I feel God’s love for me, through others.
10. I am spiritually touched by the beauty of creation.
11. I feel thankful for my blessings.
12. I feel thankful for my blessings.
13. I accept others even when they do things I think are wrong.
14. I desire to be closer to God or in union with the divine.

(possible answer for item 15 is 1 = not at all, 2 = somewhat close, 3 = very close, 4 = as close as possible)

15. In general, how close do you feel to God?

Religiosity

At present, would you call yourself a believer?

1. Yes, I am a member of a church or religious society.
2. Yes, but I am not a member of a church or religious society.
3. No
4. No, I am a convinced atheist.

II. Sample details

Table 1 Sociodemographic information about the April 2020 sample, compared to the 2016 representative sample of the Czech population.

**Characteristic**	**2016 national Czech representative sample**	**April 2020 sample**
Age (median)	*46.4*	*47.5*
Female (%)	51.3	*48.5*
Married/partner relationship (%)	59.4	*66.5*
Secondary school with graduation (%)	47.4	*32.1*
Employee/self-employed (%)	62.0	*55.0*
Believer (%)	*29.5*	*33.2*
Religiously affiliated (%)	*9.4*	*8.6*
Total	***1800***	***1273***
